# From peptides to patterning: Redox control of the master regulator PLT2 in Arabidopsis roots

**DOI:** 10.1093/plphys/kiaf264

**Published:** 2025-06-19

**Authors:** Anna Moseler, Blanca Jazmin Reyes-Hernández

**Affiliations:** Assistant Features Editor, Plant Physiology, American Society of Plant Biologists; INRES-Chemical Signalling, University of Bonn, Bonn 53117, Germany; Assistant Features Editor, Plant Physiology, American Society of Plant Biologists; Faculty of Science, Department of Plant and Environmental Sciences, Section for Plant Glycobiology, University of Copenhagen, Frederiksberg C 1871, Denmark

Root systems are essential for anchorage and for the uptake of water and nutrients. Their growth is tightly regulated through coordinated processes such as sustained cell proliferation in the meristematic zone and rapid cell elongation in the elongation zone. Root growth plasticity depends on the balance among these zones and their third developmental zone, the differentiation zone, where cells reach their final size and acquire specialized functions. A key mechanism underlying the coordination of root growth and differentiation is the precise regulation of specific target genes through both activation and repression, a process largely mediated by the PLETHORA (PLT) transcription factors, particularly PLT1 and PLT2, that establish spatial expression gradients across root zones (Aida et al. 2004; Santuari et al. 2016). In recent years, small peptides (typically fewer than 20 amino acids) have emerged as key signaling molecules in root development, acting in a hormone-like manner to modulate these transcriptional programs (Matsubayashi 2014).

A major breakthrough in understanding how PLT gradients are formed came with the discovery of the secreted peptides Root Meristem Growth Factors (RGFs; also referred to as GOLVEN or CLE-like), acting upstream of PLT transcription factors (Matsuzaki et al. 2010). Ten years later, the biochemical connection between RGFs and PLTs was elucidated, establishing that their interaction is mediated through a specific signaling mechanism. RGF1 modulates the balance between the reactive oxygen species (ROS) superoxide (O_2_⁻) and hydrogen peroxide (H_2_O_2_) in the root developmental zones. O_2_⁻ accumulates primarily in the meristematic zone, while H_2_O_2_ accumulates mainly in the differentiation zone; changes in this distribution in turn alter the stability of PLT2 (Yamada et al. 2020). However, the mechanism behind PLT2 stabilization remained unclear. Hsiao et al. (2025) now show that RGF1 signaling-dependent sulfenylation of a cysteine (Cys) in PLT2 is crucial for PLT2 stability.

PLT1 and PLT2 act redundantly to maintain root growth. While *plt1* and *plt2* single mutants exhibit slight meristem shrinkage, the *plt1 plt2* double mutant shows a pronounced decreased root meristem (Aida et al. 2004). When wild-type and *plt1* plants are treated with RGF1, the meristem expands but not in *plt2*. Using PLT-GFP lines, Hsiao and colleagues showed that RGF1 treatment increases the PLT2 distribution at the root tip, while PLT1 remains limited and slightly decreases. This indicates that PLT2 is the major contributor to the RGF1 signaling pathway.

In the next step, the researchers investigated how the ROS modulated by RGF1 impact PLT2. Since thiol (−SH) groups are common ROS targets, the authors replaced one or both Cys residues at positions 112 and 212 by serine. They complemented the *plt2* mutant with the respective C112S, C212S and C112S/C212S PLT2 variants and observed their root responses to RGF1. The gPLT2^C212S^-GFP and gPLT2^C112S^/^C212S^-GFP expressed in *plt2* lines showed broader PLT2 localization and larger meristematic zones, even without exogenous RGF1 application, highlighting a pivotal role of the 212th Cys for PLT2 stability. Monitoring ROS distribution in gPLT2^C212S^-GFP lines showed that RGF1 increased O_2_⁻ and decreased H_2_O_2_, especially at the boundary between the meristematic and elongation zones. Substituting the 212th Cys enhances PLT2 stability under oxidative conditions, suggesting that the redox state of the 212th Cys is crucial for its stability and function (Fig.). Reverse transcription quantitative PCR confirmed unchanged PLT2 transcript levels, while immunoblots revealed marked protein levels increased in the C212S lines after RGF1 treatment. Using a nonmobile PLT2-3xGFP line, the authors still observed expanded localization, excluding any effect due to PLT2 cell-to-cell movement. Together, these data demonstrate that Cys212 is essential for ROS-dependent PLT2 stabilization, indicating that the effect of RGF1 on ROS acts upstream of PLT to promote meristem expansion.

**Figure.**
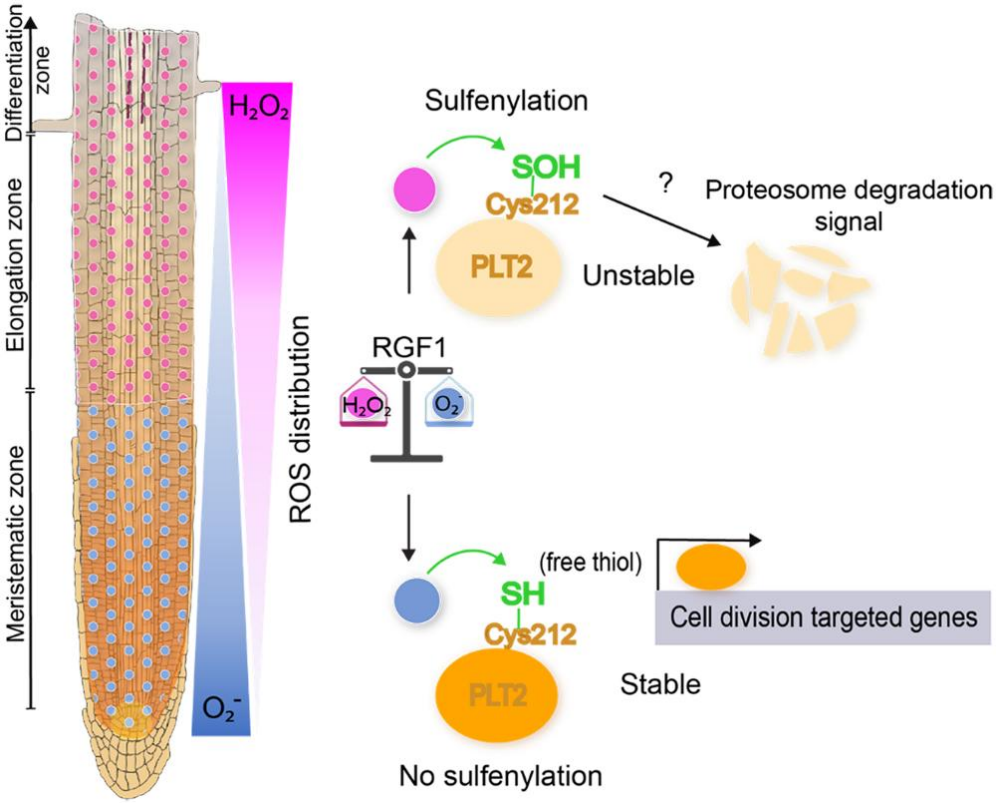
Model of RGF1-ROS signaling required for root meristem size control. RGF1 signaling regulates root meristem size through the modulation of ROS. RGF1 promotes the accumulation of hydrogen peroxide (H_2_O_2_) in the elongation zone, which triggers the sulfenylation of the 212th Cys (Cys212) on the PLT2 protein. This modification destabilizes PLT2, allowing cells to enter an elongation state and subsequently differentiate. Concurrently, RGF1 leads to the accumulation of superoxide (O_2_⁻) in the meristematic zone, creating a reducing environment that maintains Cys212 in its free thiol form (decreased sulfenylation), thereby stabilizing PLT2. This stabilization enables the accumulation of PLT2 in the meristematic zone, where it promotes the expression of genes involved in cell proliferation and represses those associated with elongation and differentiation, thus delaying the onset of maturation. The redox state of Cys212 is essential for establishing the PLT2 concentration gradient, which is shaped by RGF1 through spatial control of ROS distribution along root developmental zones (modified after Hsiao *et al.* (2025)).

The *plt2* mutant expressing either gPLT2-GFP or the corresponding C212S variant were further used to analyze the GFP signal intensities in different developmental zones. The authors detected a more stable signal for gPLT2^C212S^-GFP in the elongation zone, where higher H_2_O_2_ levels were detected, compared to the gPLT2-GFP. Additionally, the team showed that after exogenous application of H_2_O_2_, the stabilization of PLT2 leads to an increase in root meristem size, highlighting that H_2_O_2_ mainly targets the 212th Cys to modulate PLT2 stability. Generally, the reaction of H_2_O_2_ typically oxidizes thiols to sulfenic acid, but excess of H_2_O_2_ can cause overoxidation to sulfinic or sulfonic acid, leading to protein degradation. To detect specific modifications of 212th Cys of PLT2 in vivo, the authors isolated PLT2-GFP and found that this residue was more sulfenylated after H_2_O_2_ treatment, indicating that H_2_O_2_ promotes sulfenylation to modulate PLT2 stability.

The authors then examined how the C212S variant affects target gene expression. RNA-seq analysis of root tips revealed over 8,500 differentially expressed genes (DEGs) in the gPLT2-GFP line compared to the *plt2* mutant and over 10,500 DEGs in the gPLT2^C212S^-GFP line, indicating that the PLT2^C212S^ variant has a stronger impact on downstream gene regulation than the wild-type PLT2. Comparison with a previous root developmental zone–specific transcriptome analysis (Li et al. 2016) revealed enhanced modulation of zone-specific genes by the PLT2^C212S^ variant: 883 out of 1,098 genes were upregulated in the meristematic zone, and 339 of 377 genes were downregulated in the elongation zone. Gene ontology (GO) analysis showed enrichment of *cell proliferation-related terms* among upregulated genes and *cell differentiation* and *cell wall biogenesis* among the downregulated genes. These data indicate that the stabilization of PLT2 amplifies its transcriptional regulation, potentially shifting the balance toward meristem activity over differentiation.

In summary, this work confirms previous studies showing the role of the RGF1 peptide in regulating PLT2 protein stability through modulation of ROS levels during root meristem development and further demonstrates that PLT2 protein stability is controlled post-translationally by sulfenylation of the 212th Cys residue by H_2_O_2_, thereby limiting its spread in the elongation zone. Further studies are needed to unravel how individual cells perceive and integrate the concentration-dependent information provided by RGFs to generate precise root meristem patterning during root development. Another important question is how the sulfenylation of PLT2 induces the degradation of the protein. Furthermore, new tools like genetically encoded sensors for H_2_O_2_ can help to further dissect H_2_O_2_ changes dynamically and at high resolution (Nietzel et al. 2019). Nevertheless, the knowledge obtained in this study can support the development of strategies to enhance root development in adverse environmental conditions. For instance, crops expressing modified versions of PLT2 that are less sensitive to oxidative degradation could maintain root function during prolonged stress, potentially helping sustain plant productivity.

## Data Availability

No new data included in this article.
